# An insulin-like peptide specific for a cockroach male reproductive gland

**DOI:** 10.1371/journal.pone.0329852

**Published:** 2025-08-19

**Authors:** Viviana Pagone, David Pujal, José Luis Maestro

**Affiliations:** Institute of Evolutionary Biology (CSIC-Universitat Pompeu Fabra), Passeig Marítim de la Barceloneta 37-49, Barcelona, Spain; University of Leipzig Faculty of Life Sciences: Universitat Leipzig Fakultat fur Lebenswissenschaften, GERMANY

## Abstract

The insulin-IGF-signalling (IIS) pathway plays crucial roles in animal physiology. In insects, this pathway uses multiple ligands, insulin-like peptides (ILPs), and a smaller number of insulin receptors (InRs). Consequently, much of the regulation of IIS pathway activity is driven by the expression of ILPs, its timing, tissue specific expression, and mode of action: endocrine, paracrine, or autocrine. In the adult male of the cockroach *Blattella germanica*, tissue expression and the regulation in relation to food restriction of the ILPs *BgILP1–7* is similar to that observed in females. However, we identified the expression of *BgILP8*, an ILP absent in females, in the conglobate gland of males and only in this organ. *BgILP8* expression in the conglobate gland is high during the initial days of adulthood and is reduced under starvation. The conglobate gland is a male sexual gland specific to cockroaches, involved in spermatophore formation. It consists of branches of coiled tubules that merge into a main collecting tubule, which opens into the ejaculatory duct. Each tubule is lined with a layer of secretory cells, each of them traversed by small ductules made of cuticle and actin fibers, through which the secretions are released. Decreasing BgILP8 levels through RNAi resulted in a reduction in the size of secretory cells, although the total protein extracted remained unaffected. A comparison of the transcriptomes of control and BgILP8-depleted glands revealed only small differences. Further, comparing genes expressed in the conglobate gland to those expressed in adult females provided a list of putative conglobate gland-specific genes. Our results suggest that BgILP8 plays a role in the development of the conglobate gland. However, a potential function in other male organs or its possible transfer to the female in the copulation cannot be ruled out.

## Introduction

The effective transfer of viable and healthy sperm from male to female insects is essential for successful sexual reproduction. To achieve this, insects have evolved a variety of reproductive strategies. In most species, secretions from the male accessory sex glands serve multiple functions, including sperm protection, storage and activation, and hormone transfer. Furthermore, even after the sperm has been transferred to the females, these secretions can influence sperm competition, decrease female attractiveness to further mating, affect female fecundity, regulate oviposition, and protect laid eggs [1].

**Fig 1:**
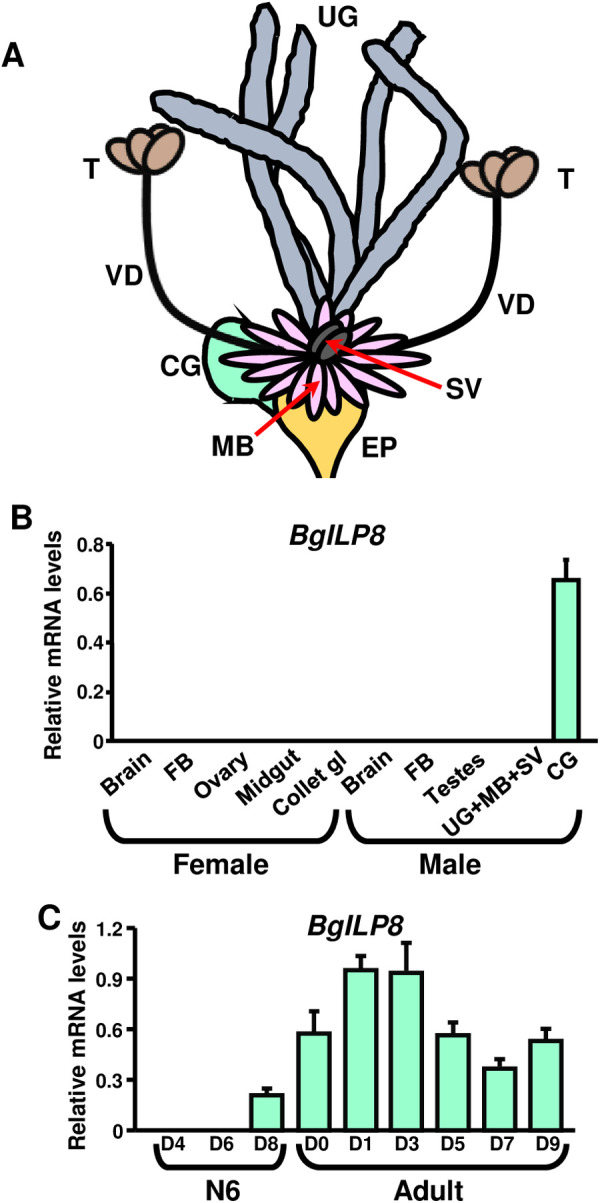
Reproductive system of the *Blattella germanica* male and *BgILP8* expression. **(A)** Schematic representation of the different organs conforming the male reproductive system. CG: conglobate gland; EP: ejaculatory pouch; MB: mushroom-shaped body; SV: seminal vesicles; T: testicle; UG: uricose glands; VD: vas deferens. **(B)** *BgILP8* mRNA levels in different *B. germanica* organ and tissues of 5-day-old adult females and males. **(C)** Conglobate gland *BgILP8* mRNA levels at different days during the last (sixth) nymphal instar (N6) and adult period. *Y*-axes indicate copies per copy of *Actin 5C*. The results are expressed as the mean ± **S.**E. **M.** (n = 3-4). FB: fat body; Collet gl: colleterial glands.

The morphology of the male insect accessory sex glands is highly diverse. In the German cockroach, *Blattella germanica*, they consist of three main structures: the mushroom-shaped body, the uricose glands, and the conglobate gland [2]. The mushroom-shaped body is a group of about 200 heterogeneous utricles arranged in the appearance of a pompom, inserted in the ejaculatory pouch and surrounding a pair of seminal vesicles (Fig 1A) [3]. The uricose glands are 4–6 opaque white tubules that run anteriorly in the male’s abdomen. They are inserted close to the insertion point of the tubules of the mushroom-shaped body and are primarily responsible for the accumulation and excretion of uric acid (Fig 1A) [3,4].

The conglobate gland, also called phallic gland, is a gland specific to male cockroaches. Its rather flat structure is made up of coiled tubules derived from a single collector tube that opens into the terminal part of the ejaculatory duct (Fig 1A) [3]. Total protein content rapidly and steadily increases from adult emergence until around day 5, after which the increase is more moderate. This is stimulated by juvenile hormone (JH), since allatectomy reduces protein accumulation and JH treatment restores it [5]. Conglobate gland function is related to spermatophore formation [5]. In the spermatophore, the sperm is packed together with secretory materials from the male accessory sex glands prior to its transfer to the female.

The insulin/insulin-like growth factor signalling (IIS) pathway regulates key processes in metazoans. In *B. germanica*, the IIS pathway is involved in the regulation of growth, reproduction, and metabolism [6–9]. The pathway is activated by the binding of ligands, in insects usually called insulin-like peptides (ILPs), to a tyrosine kinase-like membrane receptor, the insulin receptor (InR). This binding triggers a series of protein phosphorylations that will produce a set of effects at the cellular level, including changes in gene expression [10].

Although most insects have two InR, in the Polyneoptera branch, which includes cockroaches, termites, and stick insects, a second gene duplication increased the number to three [11,12]. In the cockroach *B. germanica*, all three InR are involved in the same processes, with an effect proportional to their expression levels [13].

Regarding the ILPs, the expression of 7 ILPs of *B. germanica* (*BgILP1*–*7*) has been described in different tissues of the adult female [14]. Their expression is regulated differentially depending on the nutritional status, and there is a compensatory regulation that causes the expression of one of the ILPs to increase when that of another decreases and *vice versa* [14]. It has also been shown that their expression responds differently to JH levels [15].

Recently, an analysis of transcriptomes from different species of arthropods, described the occurrence of the different sequences of molecules belonging to the insulin family [16]. In the case of *B. germanica*, the study revealed the presence of a *BgILP8* in transcriptomes identified as ‘reproductive organs and fat body of males’ [16].

In the present work, we aim to characterize the expression of *BgILP1–8* in male *B. germanica* tissues. We have determined that *BgILP8* is expressed in the conglobate gland. We have then described the structure of this gland, studied the genes that expresses and tried to identify the function of BgILP8, a singular ILP in a singular organ. Our main hypothesis is that *BgILP8* function will be related to conglobate gland physiology and/or development. However, an endocrine effect on some other tissues and organs, or even a transfer to the female into the spermatophore, cannot be discarded.

## Materials and methods

### Insects

Specimens of *B. germanica*, *Periplaneta americana,* and *Blatta orientalis* were obtained from colonies maintained in the dark and reared on dog food and water at 29 ± 1 °C and 60–70% relative humidity. Dissections were carried out in saline on carbon dioxide-anesthetized virgin males. After dissection, tissues were immediately frozen in liquid nitrogen and stored at −80˚C. For the starvation assays, males received only water after the imaginal moult and dissections were performed at adult day 5 or day 7.

### RNA extraction, cDNA synthesis, quantitative real-time PCR analysis and protein quantification

Total RNA was extracted using the HigherPurity^TM^ Tissue Total RNA Purification Kit (Canvax Biotech). cDNAs were synthesized from total RNA using the Transcriptor First Strand cDNA Synthesis kit (Roche). In the case of fat body, ovary, and midgut, we used 1 µg of total RNA for cDNA synthesis, whereas for other tissues, we concentrated the sample by lyophilization and used the total amount of the RNA. The absence of genomic contamination was confirmed using a negative control without reverse transcription. Quantitative real-time PCR analyses were carried out as previously described [15]. Primer sequences used to quantify *BgILP1*–*7*, and *Actin 5C* (used as a reference gene) have already been reported [8,14,17]. Primers for the quantification of *BgILP8* are indicated in S1 Table. All samples were run in triplicate or duplicate.

For protein quantification, conglobate glands were homogenized in PBS, pH 7.4, and centrifuged to eliminate cellular debris. Total protein was then quantified using the Bio-Rad protein assay dye reagent and bovine serum albumin as standard.

### RNA interference

A 188 bp fragment of the *BgILP8* cDNA was cloned and used to synthesize the dsRNA using MEGAscript^TM^ RNAi kit (Invitrogen) (primers in S1 Table). Control animals were treated with a 307 bp fragment of a heterologous dsRNA of the polyhedrin of *Autographa californica* nucleopolyhedrovirus. 2 µl of dsRNA at a concentration of 1 µg/µl were injected into the abdomen of day 7 sixth (last) male instar nymphs (N6D7) using a 5 µl Hamilton® 75N syringe. Animals moulted to adults approximately 2 days later. Dissections were performed on different days during the adult period.

### Microscopy

Conglobate glands of different ages and treatments were dissected and immediately fixed with 4% paraformaldehyde in PBS 0.2 M pH 6.8 for one hour. Samples were then washed with PBT (PBS pH 6.8, 0.2% Tween-20) and incubated for 20 min with a solution of 300 ng/ml phalloidin-TRICT (Sigma) for actin-F staining, washed with PBT, and then incubated for 5 min with 1 µg/ml of DAPI (Sigma) for DNA staining. The glands were mounted in Mowiol (Calbiochem) and observed using a Zeiss AxioImager Z1 microscope (Apotome) (Carl Zeiss MicroImaging).

To determine the areas of the glands, their contours were traced in microscopy images and the areas were quantified using ImageJ. To determine the size of the secretory cells, we measured the maximal distance between the cuticle at the border of the lumen and the apical part of the cell, using the ZEN 2.3 lite software (Zeiss). Only the cells from the lobes in the external part of the glands were measured to avoid the compaction and deformation of the internal lobes.

### Library preparation and analysis

Total RNA from 7-day-old control or dsILP8 conglobate glands was extracted using the HigherPurity™ Tissue Total RNA Purification kit (Canvax). Four replicates of each treatment, approximately 900 ng per sample, were processed. Samples were analyzed by 2100 Bioanalyzer (Agilent Biotechnologies) to determine RNA integrity.

Libraries were prepared at Macrogen Inc. (Republic of Korea) with the TruSeq Stranded mRNA Library Prep Kit following the TruSeq Stranded mRNA Reference Guide #1000000040498 v00 protocol from Illumina and sequenced using the Illumina NovaSeq 6000 platform (two runs of paired end sequencing x 150 cycles). The obtained mRNA-seq libraries are publicly available at the Gene Expression Omnibus repository [18], under the accession code GSE293087. Library quality was assessed with FastQC (version 0.11.7) [19]. Adapter sequences and low-quality bases on the reads ends were trimmed using Trimmomatic (version 0.39; Sliding Window:4:20) [20]. Reads shorter than 36 bp were dropped to produce the trimmed data. The trimmed libraries were aligned to the *B. germanica* genome assembly (NCBI Acc num: PRJNA203136, version 1.1) [21] using HISAT2 (version 2.1.0) [22]. Gene abundances were calculated using the *featureCounts* function implemented in the R package Rsubread (version 2.12.3, parameters: countMultiMappingReads = T, fraction = T, useMetaFeatures = T) [23], and values were normalized using the Transcript Per Million (TPM) method.

Extremely infrequent genes with zero raw counts in more than four libraries were filtered out. Differential expression analysis between dsILP8 libraries and controls was conducted with DESeq2, setting a Fold Change threshold of log_2_FC > 1 or <−1 and a False Discovery Rate (FDR) adjusted p-value threshold of 0.05 [24]. Volcano plots were generated using the R package EnhancedVolcano (version 1.16.0).

The occurrence of a signal peptide at the N-terminus of the obtained protein sequences was determined using SignalP 5.0 [25].

### Statistical analysis

All data were expressed as mean ± standard error of the mean (S.E.M.). Statistical analyses were performed using IBM SPSS Statistics 24. The comparison of results from control and treated animals was performed using Student’s *t*-test.

## Results

### Expression of *BgILP8* and other ILPs in *Blattella germanica* males

Using the *BgILP8* sequence reported by Veenstra [16] in transcriptomes from reproductive organs and fat body from adult *B. germanica* males, we designed primers to quantify *BgILP8* expression by qPCR. *BgILP8* expression was checked by qPCR in the brain, fat body, midgut, ovary, and colleterial glands of adult females, and brain, fat body, testicles, uricose glands + mushroom-shaped body + seminal vesicles dissected together, and conglobate glands of adult males. Results showed that *BgILP8* was only expressed in the male conglobate gland (Fig 1B). We also cloned the whole *BgILP8* sequence from conglobate gland cDNA (Accession Number: PQ565705; S1 Fig). BgILP8 has a 21 amino acid signal peptide and a structure corresponding to an insulin-like peptide (S1 Fig). We then analysed the *BgILP8* expression in conglobate glands of insects at different days of the sixth (last) nymphal instar (N6) and adult. The conglobate gland could not be found in N6 day 2 (N6D2) males. Only at N6D4, the first rudiments of the gland could be detected, which became somewhat more apparent on day 6. At N6D8, about one day before the imaginal moult, its morphology is already similar, although smaller, to that of the adult. *BgILP8* mRNA levels are below the detection limits at N6D4 and N6D6 and are low at N6D8. They then increase in the adult stage and are high at days 1 and 3, showing a posterior decrease at days 5, 7, and 9 (Fig 1C).

**Fig 2:**
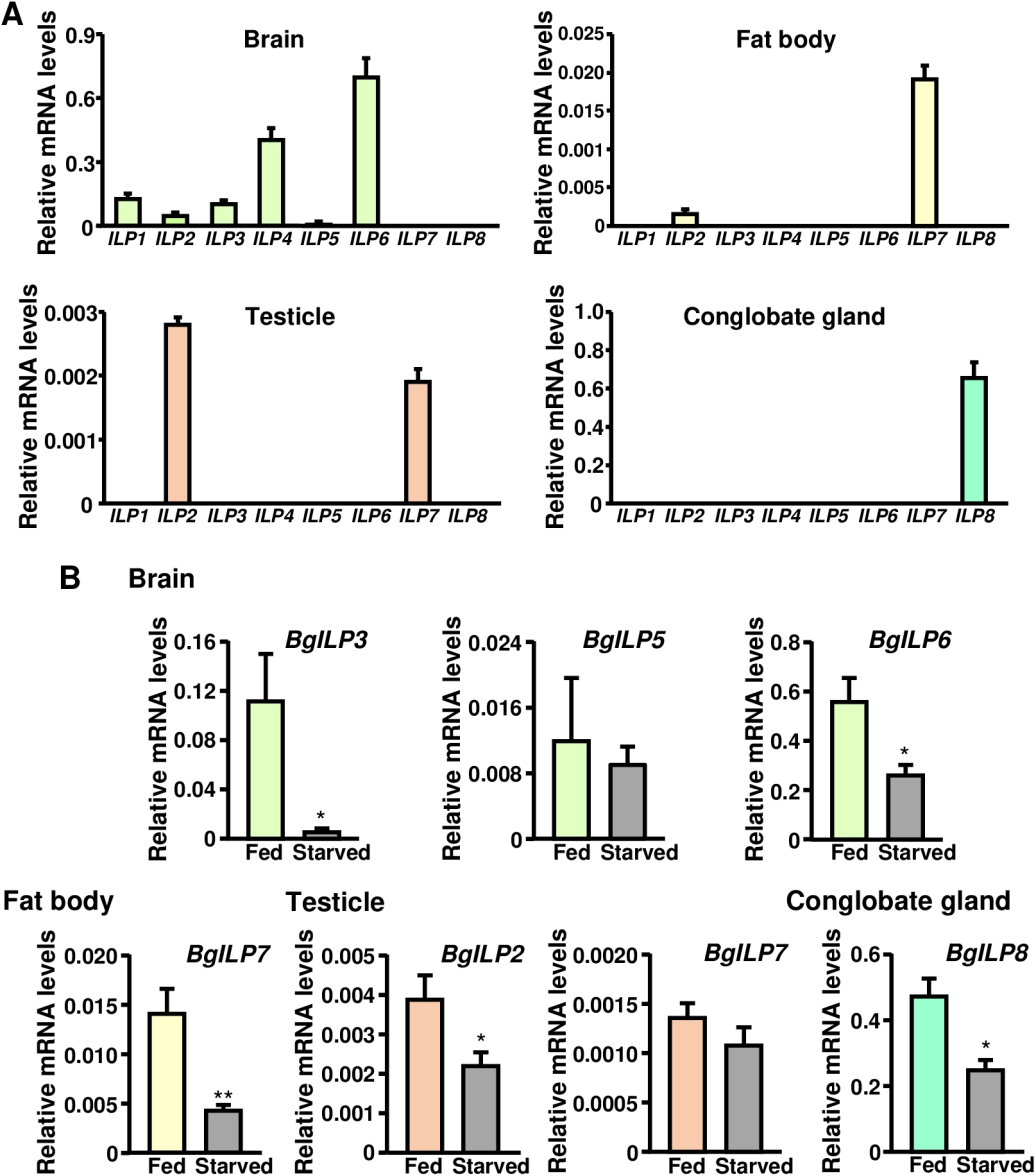
*BgILP1-8* expression in different *Blattella germanica* adult male tissues and in response to starvation. **(A)** *BgILP1-8* mRNA levels in brain, fat body, testicles, and conglobate glands of 5-day-old adult males (n = 3-4). **(B)** *BgILP3*, 5 and 6 mRNA levels in brain (n = 5-7); *BgILP7* mRNA levels in fat body (n = 4); *BgILP2* and *BgILP7* mRNA levels in testicles (n = 4); and *BgILP8* mRNA levels in conglobate gland (n = 5-7) from fed and starved 5-day-old adult males in the case of brain, fat body and testicles and 7-day-old in the case of conglobate gland. Asterisks represent significant differences between fed and starved animals (Student’s *t*-test, *p < 0.05; **p < 0.01). *Y*-axes indicate copies per copy of *Actin 5C*. The results are expressed as the mean ± **S.**E.

We also quantified the mRNA levels of *BgILP1*–*7* in 5-day-old adult male conglobate gland, brain, fat body, and testicles. Results indicated that *BgILP8* was the only ILP expressed in the male conglobate gland (Fig 2A). In the case of the brain, we could quantify *BgILP1*, *2*, *3*, *4*, *5,* and *6* mRNA, with *BgILP6* showing the highest levels and *BgILP5* showing the lowest quantifiable levels. The fat body mainly expresses *BgILP7* and, to a lesser extent, *BgILP2*. As for the testicles, they also express *BgILP2* and *BgILP7* (Fig 2A). As in the case of females [14], starvation reduced mRNA levels of *BgILP3* and *BgILP6* in the male brain, and of *BgILP7* in male fat body (Fig 2B). Brain *BgILP5* mRNA levels were very low and didn’t change in starvation (Fig 2B). In the case of testicles, *BgILP7* did not change its expression while that of *BgILP2* was reduced in starved males (Fig 2B). We also compared *BgILP8* expression in conglobate glands from fed and starved adult males. In this case, we used 7-day-old males. Results showed that starvation induces a ca. 50% reduction of *BgILP8* mRNA levels (Fig 2B).

Results then showed that the male conglobate gland was the only organ or tissue among the tested ones, that expresses *BgILP8,* and that *BgILP8* was the only BgILP expressed in the conglobate gland.

### Conglobate gland morphology and structure

**Fig 3:**
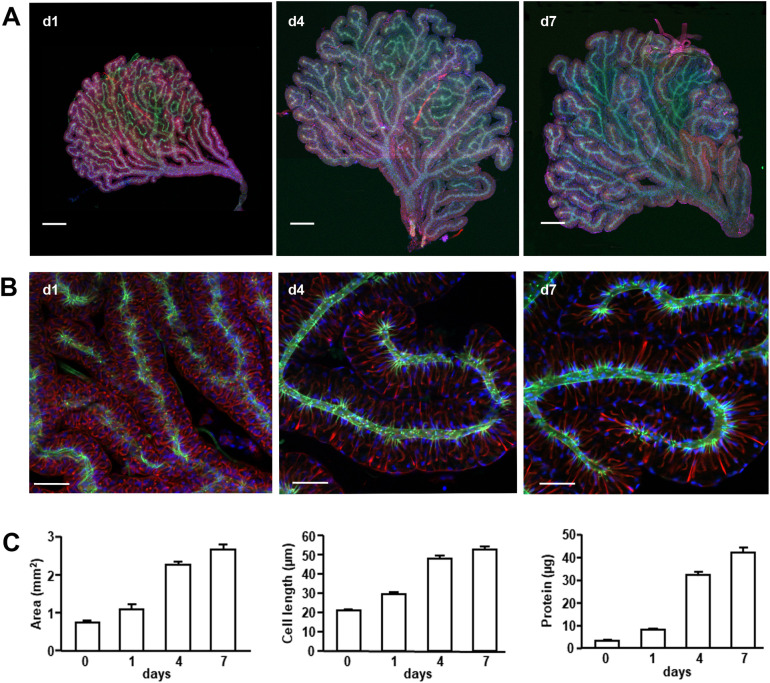
Structure and growth of the conglobate gland in *Blattella germanica.* **(A)** Conglobate glands of *B. germanica* adult males, days 1, 4, and 7. Scale bar: 200 µm. **(B)** Images of conglobate glands from the same ages at higher magnification to show the arrangement of the secretory cells along the tubules. Scale bar: 50 µm. F-actins were stained with phalloidin-TRITC (red), and nuclei with DAPI (blue). Cuticle autofluorescence is shown in green. **(C)** Total area, cell length, and total extracted protein of conglobate glands from animals of the same ages. The results are expressed as the mean ± **S.**E. Number of replicates: area (n = 4-6); cell length (number of replicates (glands): 3-5; number of measured cells per gland: 26-66, mean = 40.1); protein (n = 9-21).

The conglobate gland is a rather flat, fan-shaped gland, situated in a ventral position with respect to the hindgut, but a dorsal position with respect to the mushroom-shaped body and the uricose glands. It is composed of twisted tubules that ramify in different branches and converge in a general collecting tubule that opens into the ejaculatory duct (Fig 3A–3B).

Each of the gland tubules is made up of a single layer of secretory cells surrounding the lumen of the tubule, which is outlined by the cuticle autofluorescence (Fig 3A–3B). During adult development, the total area of the gland, the length of the secretory cells, and the total protein content increase steadily from the time of imaginal moult until day 7 (Fig 3C). The area, the length of the cells and the total protein are reduced in glands from starved animals (S2 Fig).

**Fig 4:**
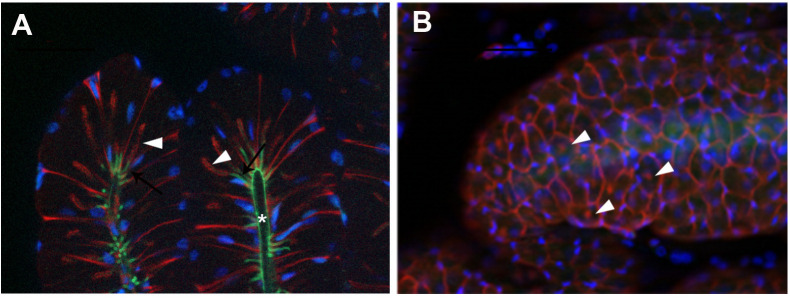
Cellular structure of the conglobate gland in *Blattella germanica.* **(A)** High-magnification image of the end of two tubules from a 7-day-old conglobate gland. The cuticle (green autofluorescence) surrounding the lumen of the tubule can be seen, which enters the secretory cell, forming a small ductule (arrow). Shortly after entering the cell, the green staining diminishes, and F-actin staining (red) surrounds the ductule and extends it into the cell (arrowhead). **(B)** Surface view of a 7-day-old conglobate gland. The F-actin (red) shows the polygonal shape of the cells, with the nuclei (blue) positioned laterally. The F-actin ductule seen entering the secretory cells in A is now observed in a central position (arrowhead). F-actins were stained with phalloidin-TRITC (red), and nuclei with DAPI (blue). Cuticle autofluorescence is shown in green. Scale bars: A: 50 µm; B: 100 µm.

An invagination of the cuticle that surrounds the lumen of the tubules enters each of the secretory cells (arrows, Fig 4A). This cuticular invagination fades as it penetrates the interior of the cell and is surrounded by actin fibers that form a small ductule that extends beyond the middle of the cell. (arrowheads, Fig 4A). Seen on the surface, the cells have a polygonal shape with the nucleus attached to the side of the cell (Fig 4B). The actin ductule inside the cell can be seen in a central position of each cell (arrowheads, Fig 4B).

**Fig 5:**
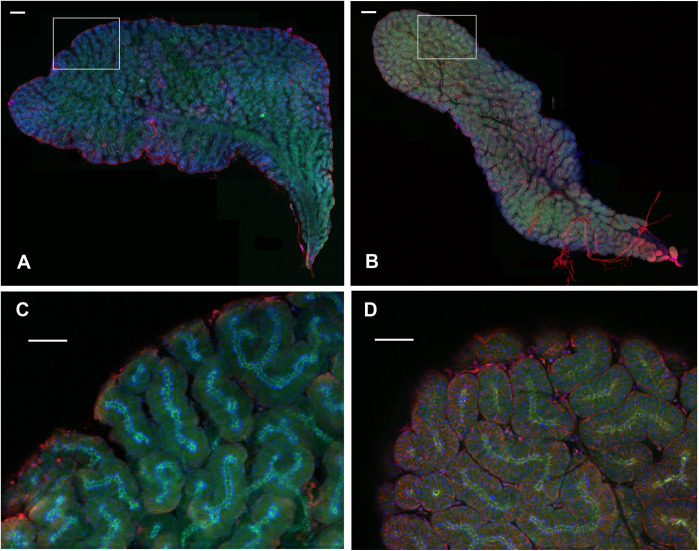
The conglobate gland of *Blatta orientalis* and *Periplaneta americana.* Glands show the same structure as in the case of *B. germanica*. A and B, conglobate glands from *B. orientalis* and *P. americana*, respectively. C and D, higher magnification of the insert to show the lobes, tubules and cellular distribution. F-actins were stained with phalloidin-TRITC (red), and nuclei with DAPI (blue). Cuticle autofluorescence is shown in green. Scale bars in A and B: 200 µm; in C and D: 100 µm.

The same morphology and organ structure are found in conglobate glands from adult males of the American cockroach *P. americana* and the oriental cockroach *B. orientalis* (Fig 5).

### BgILP8 functional studies

**Fig 6:**
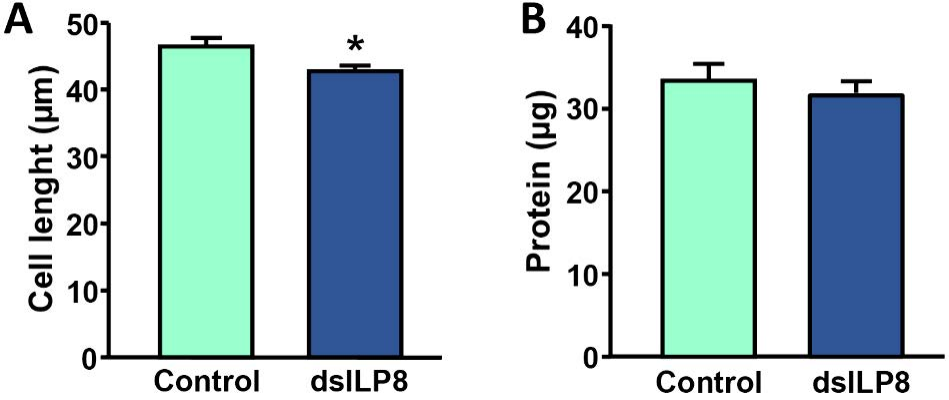
Effect of *BgILP8* RNAi on secretory cell length and protein in *Blattella germanica.* Animals on the seventh day of the last (sixth) nymphal instar (N6D7) were treated with 2 µg of dsRNA targeting *BgILP8* (dsILP8) or a heterologous dsRNA (Control). Dissections were performed at adult day 4. **(A)** Cell length of control and dsILP8 (n = 4 glands control and 4 glands dsILP8; number of measured cells per gland: 15-45, mean: 34.1). **(B)** Total proteins from extracts of Control and dsILP8 glands (n = 9-11). The results are expressed as the mean ± **S.**E. Asterisks represent significant differences between fed and starved animals (Student’s *t*-test, *p < 0.05).

In order to identify the functions of BgILP8, we depleted the corresponding mRNA levels by RNAi. Thus, we injected a non-homologous dsRNA (Control) or a dsRNA specific against *BgILP8* (dsILP8) on the seventh day of N6. We let the animals moult to adult two days later and quantified *BgILP8* mRNA levels in the conglobate gland at different days. Results showed that dsILP8 treatment produced a significant reduction of *BgILP8* mRNA by more than 80% on days 1, 4, and 5 and by 60% on day 7 (S3 Fig). The RNAi treatment produced a small (8%) but statistically significant reduction in the length of the secretory cells of conglobate glands from 4-day-old animals (Fig 6A). Although it was not statistically significant, a similar reduction was observed in cell length of glands from 7-day-old males (C: 57.06 ± 1.96 µm; dsILP8: 52.65 ± 2.36 µm, n = 5) (S4 Fig). The amount of protein extracted from the conglobate glands of 4-day-old dsILP8-treated insects, while somewhat reduced, was not statistically different from that of controls (Fig 6B).

### Analysis of conglobate glands transcriptomes

To determine the transcriptomic changes that the dsILP8 treatment produced in the conglobate glands, we repeated the treatment described above and dissected glands from 7-day-old Control and dsILP8 males, performing four replicates for each treatment. Total RNA was extracted, and mRNA libraries were constructed from each of the samples. Libraries generated on average 26.58 ± 0.59 (mean ± S.E.M.) million reads per sample and showed an overall read mapping ratio to the reference genome of 83–85% (S2 Table).

**Fig 7:**
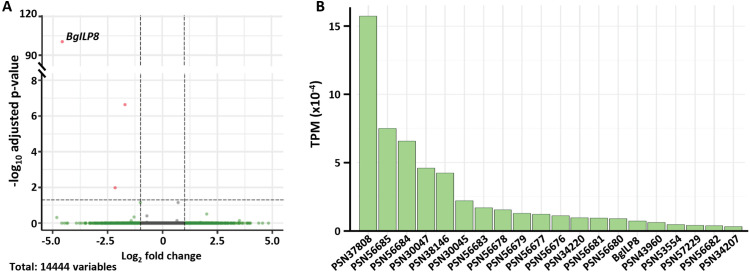
Comparative analysis of conglobate gland transcriptomics in *Blattella germanica.* **(A)** Volcano plot showing the DEG in the dsILP8 vs. control libraries. Animals on the seventh day of the last (sixth) nymphal instar (N6D7) were treated with 2 µg of dsRNA targeting *BgILP8* (dsILP8) or a heterologous dsRNA (Control). Dissections were performed at adult day 4. Red dots indicate DEG with adjusted *P*-value < 0.05 and log_2_ fold change < −1 or >1. **(B)** Transcripts per million (TPM) of the most expressed DEG from the comparison between conglobate glands control libraries and whole body 5-day-old adult female libraries (from [26]).

The comparison between Control and dsILP8 libraries under the parameters of the analysis (adjusted *P*-value < 0.05, log_2_FC < −1 or > 1) showed only three differentially expressed genes (DEG), all three downregulated in dsILP8 glands. These genes were *BgILP8* itself, *Fumarylacetoacetase*, which is involved in tyrosine and phenylalanine catabolism; and PSN52889, which a BLAST to the NCBI protein database labelled as AP-3 complex subunit beta-1, involved in intracellular vesicle traffic (Fig 7A; S3 Table).

Furthermore, to identify the genes specific to the male conglobate gland, a comparison was made between the genes found in the control libraries of conglobate glands and libraries from whole body 5-day-old adult females (n = 2) (from [26]). To perform this analysis, we filtered the results so that only genes that, on average, had more than 50 counts across the four conglobate gland libraries and those that had less than 10 counts across the two female libraries were selected. Next, we kept only the genes that had more than 10 counts in at least two libraries. This was done to filter out extremely variable genes that have a really high expression in one sample and are absent in the others. A comparison of expression levels between the two types of libraries was then performed. The results showed 101 differentially expressed genes (DEGs) whose expression is specific (or enriched) in the conglobate gland. We analyzed these genes by looking at their annotation and performing a BLAST of the unannotated genes (S4 Table). Among the genes with more than 3,000 TPMs (20 genes), we found *BgILP8*, ten serine carboxypeptidases, and three genes identified as allergen proteins (Fig 7B, S4 Table).

To screen the DEGs coding for proteins synthesized in the conglobate gland and potentially excreted into the spermatophore, the presence of signal peptides in the proteins encoded by conglobate gland DEGs was analyzed [25]. The analysis showed that 17 of these DEGs had a signal peptide, including BgILP8, 3 putative serine carboxypeptidases, and one putative neuropeptide G protein-coupled receptor (S4 Table). We could also find two proteins from the C-lectin/ hemolymph lipopolysaccharide-binding protein family (S4 Table). In addition, one of these proteins (PSN53417) showed repetitive sequences Gly-Gly-Gly-His/Tyr, Lys-Val-Pro and Pro-Val.

## Discussion

The critical role of the IIS pathway in organismal physiology necessitates precise regulatory control of its activity. In insects, the presence of a greater number of ligands (ILPs) compared to receptors suggests that pathway regulation is primarily achieved through regulation of ILP production at different moments, in different tissues and in response to different stimuli [13,14,27].

BgILP8 is a *B. germanica* insulin-like peptide that was reported [16] after our work that analysed the expression and regulation of ILPs in adult females [14]. In the present work, we uncover that *BgILP8* expression is restricted exclusively to the male conglobate gland, among the many male and female tissues and organs examined. This must be the reason why we didn´t identify *BgILP8* expression in our previous work with females [14].

The expression of *BgILP1*–*7* in the brain and fat body of *B. germanica* adult males closely resembles that observed in females [14], with the exception that in males the expression of *BgILP5* in the brain is extremely low. This suggests a potential specific effect of *BgILP5* on female physiology, although such an effect could not be demonstrated previously [14]. As for the sexual organs, while the ovary expresses exclusively *BgILP2* [14], the testicles express both *BgILP2* and *BgILP7*. On the other hand, the conglobate gland, only expresses *BgILP8*.

The expression results of different ILPs in the brain and fat body of fed and starved adult *B. germanica* males indicated that, in the same way as in females, IIS signals nutritional status and does so through the same peptides [14]. In the case of *BgILP2* in testes, its expression is reduced in starvation. This is the opposite of what is observed in ovaries [14], although the result is not surprising since these are organs with different physiology.

The conglobate gland is a male accessory sex gland characteristic of cockroaches. Its function is related to the spermatophore formation since the total amount of protein extracted from the conglobate gland and the different electrophoretic protein bands decrease when the spermatophore begins to form, whereas they gradually recover after copulation [5]. These proteins could be synthesized in the gland itself, but we cannot discard that some of them could also be synthesized in the fat body and transported to the gland via hemolymph, as suggested for some proteins of the male accessory glands of the grasshopper *Melanoplus sanguinipes* [28].

The conglobate gland of *B. germanica* is a flat, roughly fan-shaped structure composed primarily of secretory cells arranged in branching tubules that converge into a common tube. The general structure of the gland is similar in *B. orientalis* and *P. americana*, thus showing a high degree of conservation in organisms separated by more than 200 million years [29]. The total area, cell length, and total protein steadily increase after the imaginal moult. JH production by the *corpora allata* of adult males also follows a similar profile, at least until day 4–5 [30] and protein accumulation in the gland is stimulated by JH [5].

Ultrastructural observations of the conglobate gland of *P. americana* show that the lumen of the gland is lined by a layer of cuticle, formed by epicuticle, exocuticle and endocuticle, coming from an invagination of the integument [31]. In that study, they also observed the continuity in the cuticle penetrating the secretory cells in small intracellular ductules. Upon entering the cells, a change in the structure of the ductule occurs, which goes from a cuticle structure to a reticulated layer appearance under the electron microscope, which suggests a porous nature [31]. This reticulated layer is adjacent to the microvilli of the secretory cells [31]. In the case of *B. germanica*, we could also observe one central small tubular ductule formed by cuticle and actin fibers that enters each of the secretory cells. These actin structures may facilitate cellular secretion into the small ductules.

*BgILP8* mRNA levels in the conglobate gland are undetectable until the last days of the nymphal period and show a maximum towards days 1–3 of the adult, with some intermediate levels later. Although mRNA levels do not indicate the amount of active peptide or levels of release, the expression profile suggests a role of this peptide in the development of the gland. The reduction of *BgILP8* expression levels in starved males, which show underdeveloped glands, could also point in that direction.

RNAi-triggered depletion of *BgILP8* mRNA levels resulted in a reduction in the length of secretory cells in the conglobate gland and the occurrence of three DEG, one of them being *BgILP8* itself. The reduction in cell size is small and the effect observed in gene expression is limited. However, it should be noted that the other ILPs must be fully active in dsILP8 individuals, and could act on the conglobate gland as endocrine factors from the hemolymph. Furthermore, the effect of RNAi is a knockdown and not a knockout, and we do not know whether the remnants of BgILP8 can still maintain part of the activity and reduce the effect of the treatment.

The comparison between genes expressed in the conglobate gland with genes expressed in the female whole body provided a list of 101 genes with specific (or enriched) expression in the conglobate gland. We could not easily find orthologies when comparing the *B. germanica* conglobate gland proteins with those from accessory sex glands from other species. Although the functions of the seminal fluids can be preserved, their individual proteins tend to evolve fast and are not well conserved [32–36]. This suggests that proteins from the male accessory sex glands may be involved in sexual selection [37].

The proteins encoded by the conglobate gland DEGs that contain a signal peptide will putatively be secreted proteins in the form of membrane receptors, peptide hormones, or, in this case, spermatophore proteins prepared for their transfer to the female during copulation. Among the 17 proteins identified as containing a signal peptide, one of them is a G protein-coupled receptor. Another one is BgILP8 itself. Two of the proteins belong to the C-lectin/hemolymph lipopolysaccharide-binding protein family. In *D. melanogaster*, two C-type lectins help regulate the transfer of seminal proteins to the females, their stability, and their storage [38]. Finally, the occurrence of highly repetitive sequences in one of the signal peptide-containing proteins suggests that it can be a structural component of the spermatophore [33].

A sequence similarity tree of the ILPs from different Blattodea species, including *B. germanica*, *P. americana* and *B. orientalis* don’t show any clear ortholog of BgILP8, and only one sequence from the cockroach *Diploptera punctata* is found as a sister sequence, although with a quite high level of divergence [39]. Therefore, the occurrence of *BgILP8* in other species of Blattodea or its expression in the conglobate gland has not been proven so far.

BgILP8 appears to be involved in the development of the conglobate gland. However, we do not know whether it might also have an endocrine effect on other male tissues, or even be incorporated into the spermatophore and have an effect on the female after copulation. Preliminary experiments have not shown a reduction in the reproductive fitness of dsILP8 males, but the issue deserves a specific approach.

The results presented in this study have uncovered the expression of a singular ILP, *BgILP8*, in a singular organ, the conglobate gland, characteristic of cockroaches. What could be the reason for this acquisition? The occurrence of multiple copies of a gene, in this case the ILPs, allows for versatility in the regulation of their expression and stability of the processes they control [13,40].

## Supporting information

S1 Fig cDNA and amino acid sequence of BgILP8. The coding region in the cDNA sequence is in italics. In the amino acid sequence, the signal peptide is underlined. B-chain, C-peptide and A-chain are highlighted in blue, yellow, and pink, respectively. The characteristic cysteines of the insulin-like sequence are in red. The pair basic amino acids that provide the cleavage sites for producing the final mature protein are in green. These sites were selected as the most feasible according to Veenstra, J.A. (2000) Arch. Insect Biochem. Physiol. 43, 49–63. We also indicate with a box a putative site of processing by Furin-like enzymes that would result in cleavage after the tetrapeptide RKRR (Tian et al. (2011) Int J Mol Sci 12, 1060–1065).
(PDF)

S2 Fig Effect of starvation on *Blattella germanica* conglobate gland. Animals were starved since the imaginal moult and glands were dissected on day 7. Graphs show area (n = 4); cell length (n = 4 glands; number of measured cells per gland: 34–57, mean: 45.0); and protein (n = 9–10). Asterisks represent significant differences between fed and starved animals (Student’s *t*-test, **p < 0.005; ***p < 0.0001).
(PDF)

S3 Fig Effect of *BgILP8* RNAi on conglobate glands. Animals on the seventh day of the last (sixth) nymphal instar (N6D7) were treated with 2 µg of dsRNA targeting *BgILP8* (dsILP8) or a heterologous dsRNA (C: Control). Dissections were performed at day 1, 4, 5 and 7 of the adult period. *Y*-axes indicate copies per copy of *Actin 5C*. The results are expressed as the mean ± S.E. (day 1, n = 5; day 4, n = 10; day 5, n = 5; day 7, n = 7–12). Asterisks represent significant differences between Control and dsILP8 animals (Student’s *t*-test, *p > 0,05; ***p < 0.001).
(PDF)

S4 Fig Effect of *BgILP8* RNAi on secretory cell length. Animals on the seventh day of the last (sixth) nymphal instar (N6D7) were treated with 2 µg of dsRNA targeting *BgILP8* (dsILP8) or a heterologous dsRNA (Control). Dissections were performed at adult day 7. Cell length of control and dsILP8 (n = 5 glands; number of measured cells per gland: 4–16, mean: 9.1).
(PDF)

S1 Table Primers used for the quantification of *BgILP8* and for the synthesis of dsRNA against *BgILP8.* (PDF)

S2 Table Summary of the Control (dspolyh) and dsILP8 conglobate gland libraries analysis parameters. (PDF)

S3 Table Differentially expressed genes (DEG) in the comparison between control and dsILP8 libraries. (XLSX)

S4 Table List of genes with specific (or enriched) expression in conglobate gland compared to adult female whole body. (XLSX)
