# Supplementary material for: An insulin-like peptide specific for a cockroach male reproductive gland
Source: PLoS One. 2025 Aug 19;20(8):e0329852. doi: 10.1371/journal.pone.0329852 (PMC12364350; doi:10.1371/journal.pone.0329852)
Supplement: S1 Fig — The coding region in the cDNA sequence is in italics. In the amino acid sequence, the signal peptide is underlined. B-chain, C-peptide and A-chain are highlighted in blue, yellow, and pink, respectively. The characteristic cysteines of the insulin-like sequence are in red. The pair basic amino acids that provide the cleavage sites for producing the final mature protein are in green. These sites were selected as the most feasible according to Veenstra, J.A. (2000) Arch. Insect Biochem. Physiol. 43, 49–63. We also indicate with a box a putative site of processing by Furin-like enzymes that would result in cleavage after the tetrapeptide RKRR (Tian et al. (2011) Int J Mol Sci 12, 1060–1065). (PDF) [file pone.0329852.s001.pdf]

BgILP8 **:ACCACATAATAAGGACTTTGATT*****ATGTTTCAGATATGGAAGCTAAGTCTACTTGTGATAATGGCATAC***
TTTGGTATCGTTCTAACTGAAACGATTAATACAATGACTAATAGATACTGTGGACCAAATCTTCGCATTGAGTTAC
AAAGAGTTTGTAGCAGCAGATATAATGAGCTTAGTCGGAGGGACACGAAAGACTCTGCTGAATTTCCATTTCATTT
CGGAATTGTGAAACGGAATCCTCCATCTTTAATGAGTAGAAGATCATTCCGGAAACGTAGACAGGCTGCTGATGAA
TGCTGCACTAATAAAGGGTGTACTATAGCCGAACTTAGATCATACTGCCAAGACGATTG**ATTTATTACATATTCAT**
TTAAAAATACT

BgILP8:MFQIWKLSLLVIMAYFGIVLTETINTMTNRYCGPNLRIELQRVCSSRYNELSRRDTKDSAEFPFHFGIV
KRNPPSLMSRRSFRKRRQAADECCTNKGCTIAELRSYCQDD

Figure S1. cDNA and amino acid sequence of BgILP8. The coding region in the cDNA sequence is in italics. In the amino acid sequence the signal peptide is underlined. B-chain, C-peptide and A-chain are highlighted in blue, yellow, and pink, respectively. The characteristic cysteines of the insulin-like sequence are in red. The pair basic amino acids that would provide the cleavage sites for producing the final mature protein are in green. These sites were selected as the most feasible according to Veenstra, J.A. (2000) Arch. Insect Biochem. Physiol. 43, 49–63. We also indicate with a box a putative site of processing by Furin-like enzymes that would result in cleavage after the tetrapeptide RKRR (Tian et al. (2011) Int J Mol Sci 12, 1060-1065).
