# Supplementary material for: An insulin-like peptide specific for a cockroach male reproductive gland
Source: PLoS One. 2025 Aug 19;20(8):e0329852. doi: 10.1371/journal.pone.0329852 (PMC12364350; doi:10.1371/journal.pone.0329852)
Supplement: S2 Fig — Animals were starved since the imaginal moult and glands were dissected on day 7. Graphs show area (n = 4); cell length (n = 4 glands; number of measured cells per gland: 34–57, mean: 45.0); and protein (n = 9–10). Asterisks represent significant differences between fed and starved animals (Student’s t-test, **p < 0.005; ***p < 0.0001). (PDF) [file pone.0329852.s002.pdf]

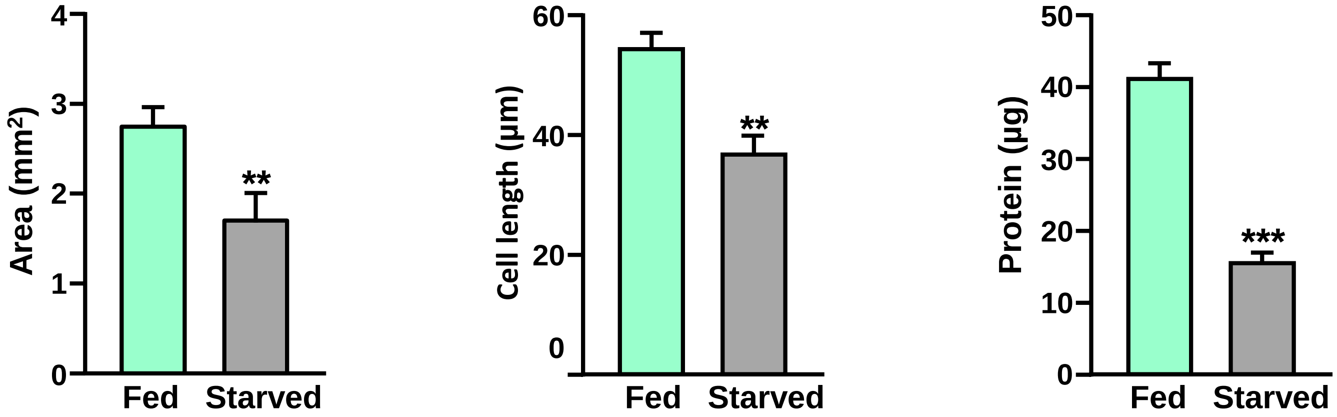

Figure S2. Effect of starvation on *Blattella germanica* conglobate gland. Animals were starved since the imaginal moult and glands were dissected on day 7. Graphs show area (n = 4); cell length (n = 4 glands; number of measured cells per gland: 34-57, mean: 45.0); and protein (n = 9-10). Asterisks represent significant differences between fed and starved animals (Student's *t*-test, $^{**}p<0,005$; $^{***}p<0.0001$).
