# Supplementary material for: An insulin-like peptide specific for a cockroach male reproductive gland
Source: PLoS One. 2025 Aug 19;20(8):e0329852. doi: 10.1371/journal.pone.0329852 (PMC12364350; doi:10.1371/journal.pone.0329852)
Supplement: S3 Fig — Animals on the seventh day of the last (sixth) nymphal instar (N6D7) were treated with 2 µg of dsRNA targeting BgILP8 (dsILP8) or a heterologous dsRNA (C: Control). Dissections were performed at day 1, 4, 5 and 7 of the adult period. Y-axes indicate copies per copy of Actin 5C. The results are expressed as the mean ± S.E. (day 1, n = 5; day 4, n = 10; day 5, n = 5; day 7, n = 7–12). Asterisks represent significant differences between Control and dsILP8 animals (Student’s t-test, *p > 0,05; ***p < 0.001). (PDF) [file pone.0329852.s003.pdf]

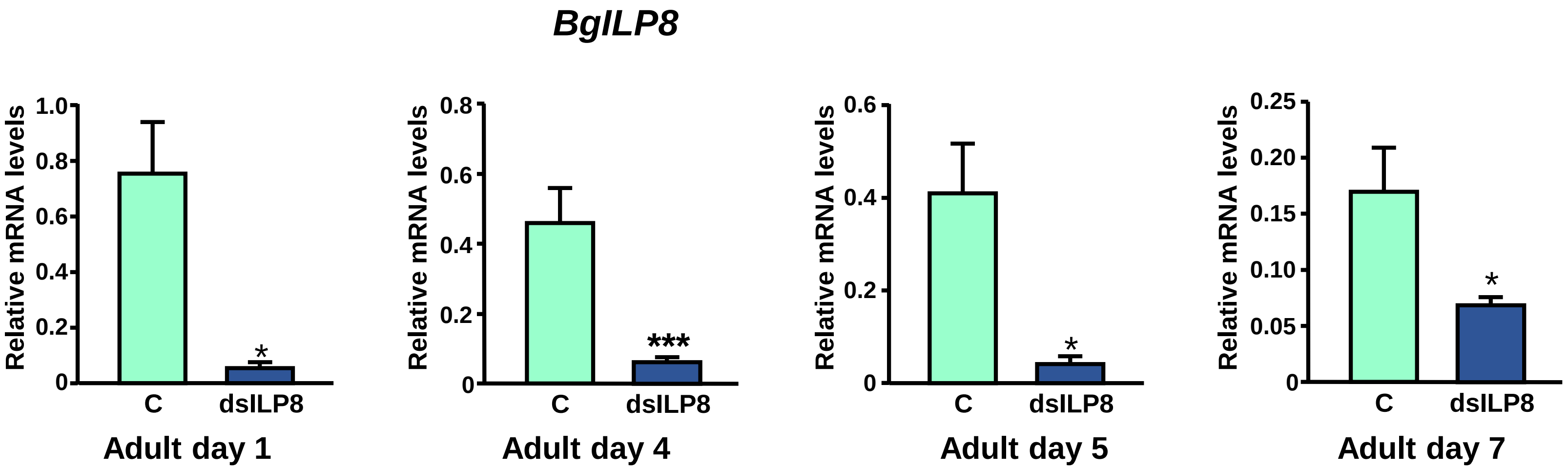

Figure S3. Effect of *BgILP8* RNAi on conglobate glands. Animals on the seventh day of the last (sixth) nymphal instar (N6D7) were treated with 2 µg of dsRNA targeting *BgILP8* (dsILP8) or a heterologous dsRNA (C: Control). Dissections were performed at day 1, 4, 5 and 7 of the adult period. *Y*-axes indicate copies per copy of *Actin 5C*. The results are expressed as the mean ± S.E. (day 1, n = 5; day 4, n = 10, day 5, n = 5, day 7, n = 7-12). Asterisks represent significant differences between Control and dsILP8 animals (Student’s *t*-test, *p>0,05; ***p < 0.001).
