# Supplementary material for: An insulin-like peptide specific for a cockroach male reproductive gland
Source: PLoS One. 2025 Aug 19;20(8):e0329852. doi: 10.1371/journal.pone.0329852 (PMC12364350; doi:10.1371/journal.pone.0329852)
Supplement: S4 Fig — Animals on the seventh day of the last (sixth) nymphal instar (N6D7) were treated with 2 µg of dsRNA targeting BgILP8 (dsILP8) or a heterologous dsRNA (Control). Dissections were performed at adult day 7. Cell length of control and dsILP8 (n = 5 glands; number of measured cells per gland: 4–16, mean: 9.1). (PDF) [file pone.0329852.s004.pdf]

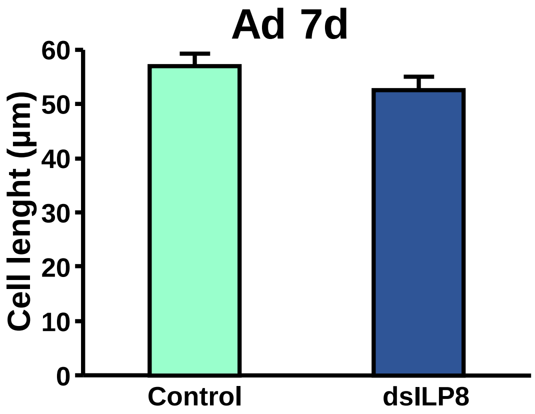

Figure S4. Effect of *BgILP8* RNAi on secretory cell length. Animals on the seventh day of the last (sixth) nymphal instar (N6D7) were treated with 2 µg of dsRNA targeting *BgILP8* (dsILP8) or a heterologous dsRNA (Control). Dissections were performed at adult day 7. Cell length of control and dsILP8 (n = 5 glands; number of measured cells per gland: 4-16, mean: 9.1).
