# Supplementary material for: An insulin-like peptide specific for a cockroach male reproductive gland
Source: PLoS One. 2025 Aug 19;20(8):e0329852. doi: 10.1371/journal.pone.0329852 (PMC12364350; doi:10.1371/journal.pone.0329852)
Supplement: S1 Table — (PDF) [file pone.0329852.s005.pdf]

Table S1. Primers used for the quantification by qPCR and for the synthesis of dsRNA against *BgILP8*.

**qPCR quantification**		
**Gene (Accession number)**	**Primer**	**Sequence**
BgILP1 (LT984754)	Fwd	AGAAGCAGAATTCCCTTTCCG
	Rv	TCATCGACAATGCCTCCGT
BgILP2 (LT971386)	Fwd	TGAATGACTCGGGCTTCTGG
	Rv	AGAGCTGACGCACTTGATCTTG
BgILP3 (LT984755)	Fwd	TGACGATTTGCTTCTCATTGTCA
	Rv	CCACCAGTTTATTCCCGCA
BgILP4 (LT984756)	Fwd	CACTGTCAGAATCCCAATCGG
	Rv	CAAATTGCATGATCTCCACCAG
BgILP5 (LT984757)	Fwd	GGCAAATTCATTGAAACCCAA
	Rv	TTCGTTGACGGAACATCCTTT
BgILP6 (LT984758)	Fwd	ACACGCCTCCCGGAAATACT
	Rv	ATTGCTTGGCCTTGGTGAAT
BgILP7 (LT984759)	Fwd	CGCCGTCATCTGGAGTTAAT
	Rv	TGTCCAGAGTGCAACCTGTC
BgILP8 (PQ565705)	Fwd	ACTGTGGACCAAATCTTCGC
	Rv	CAGCAGAGTCTTTCGTGTCC
Actin 5C (AJ862721)	Fwd	AGCTTCCTGATGGTCAGGTGA
	Rv	GTCGGCAATTCCAGGGTACATGGT

**dsRNA synthesis**			
**Gene**	**Primer**	**Sequence**	**Size (bp)**
BgILP8 (PQ565705)	Fwd	ACTCTGCTGAATTTCCATTTCA	188
	Rv	AGTATTTTTAAATGAATATGTAATAAA	
Control (K01149)	Fwd	CCTACGTGTACGACAACAAGT	441
	Rv	ATGAAGGCTCGACGATCCTA	
