# Supplementary material for: An insulin-like peptide specific for a cockroach male reproductive gland
Source: PLoS One. 2025 Aug 19;20(8):e0329852. doi: 10.1371/journal.pone.0329852 (PMC12364350; doi:10.1371/journal.pone.0329852)
Supplement: S2 Table — (PDF) [file pone.0329852.s006.pdf]

Table S2. Summary of the Control (dspolyh) and dsILP8 conglobate gland libraries analysis parameters

Library	Treatment	Total Raw Reads	Processed Reads	Mapped Reads	Mapped Reads Percentage
GC_dspolyh_1	Control	28758384	27829842	23548944	84.62
GC_dspolyh_5	Control	26633817	25783299	21566996	83.65
GC_dspolyh_6	Control	25386690	24574332	20800127	84.64
GC_dspolyh_8	Control	25663195	24837807	20633616	83.07
GC_dsILP8_2	dsILP8	28247079	27406624	22901010	83.56
GC_dsILP8_4	dsILP8	23760621	23062739	19590091	84.94
GC_dsILP8_5	dsILP8	27845060	27003296	22858048	84.65
GC_dsILP8_8	dsILP8	26333639	25333003	21499767	84.87
